# Extracellular Vesicles as a Therapeutic Tool for Kidney Disease: Current Advances and Perspectives

**DOI:** 10.3390/ijms22115787

**Published:** 2021-05-28

**Authors:** Raphael Rodrigues Corrêa, Estela Mancheño Juncosa, Rosalinde Masereeuw, Rafael Soares Lindoso

**Affiliations:** 1 Institute of Biophysics Carlos Chagas Filho, Federal University of Rio de Janeiro, Rio de Janeiro 21941-902, Brazil; raphaelrodrigues28@biof.ufrj.br; 2 Division of Pharmacology, Utrecht Institute for Pharmaceutical Sciences, Utrecht University, 3584 CG Utrecht, The Netherlands; e.manchenojuncosa@students.uu.nl; 3 National Institute of Science and Technology for Regenerative Medicine-REGENERA, Federal University of Rio de Janeiro, Rio de Janeiro 21941-902, Brazil

**Keywords:** extracellular vesicles, kidney disease, stem cell, isolation methods, bioengineering vesicles, bioreactor, 3D culture

## Abstract

Extracellular vesicles (EVs) have been described as important mediators of cell communication, regulating several physiological processes, including tissue recovery and regeneration. In the kidneys, EVs derived from stem cells have been shown to support tissue recovery in diverse disease models and have been considered an interesting alternative to cell therapy. For this purpose, however, several challenges remain to be overcome, such as the requirement of a high number of EVs for human therapy and the need for optimization of techniques for their isolation and characterization. Moreover, the kidney’s complexity and the pathological process to be treated require that EVs present a heterogeneous group of molecules to be delivered. In this review, we discuss the recent advances in the use of EVs as a therapeutic tool for kidney diseases. Moreover, we give an overview of the new technologies applied to improve EVs’ efficacy, such as novel methods of EV production and isolation by means of bioreactors and microfluidics, bioengineering the EV content and the use of alternative cell sources, including kidney organoids, to support their transfer to clinical applications.

## 1. Introduction

Stem cells have been widely investigated in preclinical and clinical studies for therapeutic purposes, including kidney disease, due to their plasticity and self-renewing and proliferative capacity [1]. The role of stem cells in the treatment of acute kidney injury (AKI) and chronic kidney diseases (CKD) has been widely described. These roles include supporting tissue repair, protecting renal architecture and preserving kidney function [2]. Increasing evidence has shown that the main mechanism involved in stem cell action is given by their paracrine capacity for secreting soluble factors and extracellular vesicles (EVs) [3]. EVs are nanosized lipid bilayer structures released by cells that participate in intercellular communication through the transfer of bioactive molecules, such as proteins, nucleic acids and lipids [4]. Unlike soluble factors, the encapsulation capacity of EVs confers high stability to the molecules, protecting them from enzymatic degradation and mediating their entrance into the recipient cell. The use of EVs presents several advantages as cell-based therapies because they are less immunogenic and present a lower risk of tumor generation. Besides, EVs have an intrinsic capacity to cross tissue and cellular barriers and display tropism to injured sites [4].

In general, EVs can be classified into three main subtypes based on their biogenesis, size and content: exosomes, microvesicles and apoptotic bodies. Exosomes are derived from the endosomal network, ranging from 30 to 100 nm in diameter, and are released to the extracellular space after fusion of multivesicular bodies (MVBs) structures with the plasma membrane [4]. Since exosome biogenesis is regulated by the endosomal sorting complexes required for transport (ESCRT) pathway, part of their composition presents proteins from ESCRT and other accessory proteins such as Alix, TSG101 and HSP90β [5]. Together with the tetraspanins CD9, CD63 and CD90, which are enriched in the exosomes, these proteins are used as biomarkers to identify and isolate exosomes [6]. The microvesicles subtype typically ranges between 100 nm and 1 μm and is released by the outward budding of the plasma membrane, carrying a group of cell surface proteins [7]. Different from the other subtypes, the apoptotic bodies are released by dying cells and range between 1 and 4 μm in size. This EV subtype can carry chromatin and organelles, presenting protein compositions similar to the cell lysate [7]. In general, in protocols used for EV isolation with therapeutic purposes this subtype is selectively removed in the initial steps.

In addition to the common molecules present in the EV subtypes, those derived from stem cells possess a unique composition of bioactive molecules responsible for their regenerative properties. This was confirmed by omics approaches to characterize the RNA and protein profiles of EVs [8,9,10,11]. Among the various molecules transferred by EVs, the microRNAs (miRNAs) have been considered critical elements in kidney cell reprogramming. Such importance has been evidenced by the abrogation of the protective effect of EVs when miRNAs were depleted from stem cells by Drosha knockdown, an endoribonuclease involved in the initial step of miRNA biogenesis [12]. Further, the EVs were found to contain proteins involved in angiogenesis, modulation of the inflammatory response, proliferation and extracellular matrix remodeling [9,10,11,13]. The diversity in the molecular profile of stem cell-derived EVs supports their complex regulatory mechanisms, acting at multiple fronts during the kidney regenerative processes.

**Figure 1:**
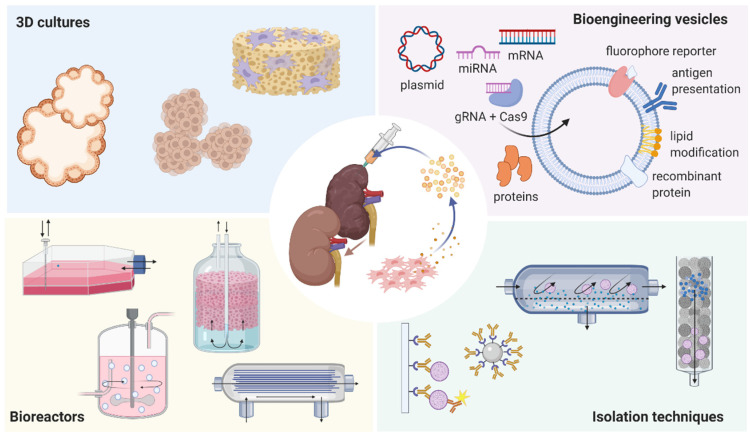
Strategies to support extracellular vesicles production for the treatment of kidney diseases. Approaches to improve the production, isolation and/or efficacy of extracellular vesicles to treat kidney diseases, which include: applications of advanced bioreactors, three-dimensional (3D) cultures such as organoids, improved isolation techniques and use of biotechnology in EV design (image created with BioRender.com (accessed on 7 May 2021)).

Despite the advances in understanding the role of EVs in cell communication and their regenerative capacity in kidney disease, their clinical application still requires further efforts. For this, new strategies are being developed, combining in-depth stem cell biology knowledge, new methods in EV isolation and changes in EV composition (see Figure 1). Here, we present an overview of the different cell sources of therapeutic EVs and show the new technologies that can be used to translate EVs into the clinic for the treatment of kidney diseases.

## 2. Cell Source of Therapeutic EVs for the Treatment of Kidney Diseases

The biological effects of EVs are given by their cargo, for which the molecular composition is directly related to the cell origin. Mesenchymal stromal cells (MSCs) represent one of the main sources of therapeutic EVs in kidney disease models [14]. Although MSCs from different tissues, such as adipose tissue, human umbilical cordon and bone marrow, share common effects in kidney recovery, the efficiency and regulatory pathways triggered by EVs vary among the MSC subtypes [15,16,17,18,19]. Therefore, recent studies have been dedicated to defining the most optimal cell source for therapeutic EVs production and how to improve its efficacy in the treatment of kidney diseases (see Table 1) [20,21,22,23,24,25,26,27,28,29,30,31,32,33,34,35,36,37,38,39,40,41,42,43,44].

### 2.1. Bone Marrow-Derived Mesenchymal Stromal Cells (BMMSCs)

BMMSCs are adult nonhematopoietic stromal cells that have been widely used as a source for therapeutic EVs in various disease models over the last years [21,27,40,45,46,47,48]. In the kidney, the EVs were shown to transfer regulatory molecules that have a fundamental role in AKI recovery and reduce CKD progression.

New strategies to improve BMMSC-EVs have focused on their biodistribution. This is an important element of their therapeutic efficacy as clearance is rapidly mediated by the innate immune system, reducing their availability for damaged tissues [49]. An interesting approach to overcome such problems was given by developing a nanofiber hydrogel enriched with BMMSC-EVs [25]. Under physiological conditions, the nanofiber hydrogel forms a three-dimensional (3D) fibrous network that allows a more sustainable release of EVs and increases tissue exposure to the BMMSC-EVs. As a result, tubular cell apoptosis was more rapidly reduced [25]. Moreover, in a different approach, serial clamping of the aorta, superior mesenteric artery, celiac trunk and renal artery drove the blood circulation to the kidney, increasing the delivery of BMMSC-EVs to the organ. Such a maneuver also reduced the spread of EVs to other organs, further enhancing kidney specificity [26].

**Table 1:** Therapeutic effects of EVs derived from different cell sources.

Cell Type	EV Population	Administration Method	Disease Model	Biological Effect	Bioactive Molecule in EVs	[Ref.]
BMMSCs	EVs	Intravenous Injection	Unilateral nephrectomy + unilateral IRI (AKI)	Enhancement of tubular cell proliferation, anti-apoptosis and reduced fibrosis in long term	-	[20]
	EVs	Intravenous Injection	Glycerol-induced AKI	Cell proliferation, support of morphologic and functional recovery	mRNAs (e.g., POLR2E, SUMO-1)	[21]
	EVs	Intravenous Injection	5/6 subtotal nephrectomy	Reduced tubular atrophy Improved kidney function	-	[22]
	EVs	Intravenous Injection	Unilateral ureteral obstruction	Improved kidney function Protection against EMT and kidney failure	miRNAs (e.g., miR-29, miR-30, miR-210-3p)	[23]
	EVs	Intravenous Injection	Glycerol-induced AKI	Impairment of morphology recovery and kidney function	miRNAs (e.g., miR-483–5p, miR-191, miR-28–3p, miR-423, miR-24)	[12]
	EVs	Intravenous Injection	Cisplatin-induced AKI	Reduction in inflammation and cell death, increased cell proliferation	-	[24]
	EVs	Renal intracapsular injection	Bilateral IRI	Decreased cell apoptosis and inflammation, endothelial cell proliferation, fibrosis reduction	-	[25]
	EVs	Intra-arterial injection	Cisplatin-induced AKI	Improved kidney function, cell proliferation, reduced inflammation	-	[26]
ADMSCs	EVs	Intravenous Injection	Cisplatin-induced AKI	Reduction in apoptosis, oxidative stress and inflammation	-	[27]
	EVs	Intravenous Injection	DOCA-salt hypertensive model	Prevention of kidney fibrosis and inflammatory response	-	[28]
	EVs	Renal intracapsular injection	Bilateral IRI	Inhibition of apoptosis, immunomodulation, recovery of intracellular ATP, preservation of mitochondria	-	[29]
	Exosomes	Intravenous Injection	Sepsis-AKI	Improved kidney function, reduced inflammatory cytokines release, reduced mortality	-	[30]
	EVs	Intra-arterial injection	Unilateral renal stenosis	Increased cell proliferation, angiogenesis, immunomodulation	Senescence-associated miRNA (e.g., miR-222-3p, miR-143-5p)	[31]
	EVs	Intra-arterial injection	Unilateral renal stenosis + metabolic syndrome	Reduced inflammation, improved medullary oxygenation, reduced fibrosis	IL-10, TGF-β	[9,32]
EPCs	EVs	Intravenous Injection	Sepsis-AKI	Reduced inflammation and apoptosis	miR-93-5p	[33]
KPCs	EVs	Intravenous Injection	Unilateral IRI + unilateral nephrectomy	Amelioration of kidney function, reduced ischemic damage	miRNAs (e.g., miR-299-5p, miR-23a-3p, miR-302b-3p)	[34]
HLSCs	EVs	Intravenous Injection	Diabetic nephropathy	Prevention and reversal of the progression of glomerular and interstitial fibrosis	miRNAs (e.g., miR-146a-5p, miR-17-5p, miR-106a-5p, miR-155-5p)	[26]
	EVs	Intravenous Injection	Glycerol-induced AKI	Improved kidney function and cell proliferation, reduced tubular necrosis	-	[35]
PDMSCs	EVs	Intravenous Injection	Bilateral IRI	Reduced inflammation, inhibited cell apoptosis, antioxidant effects	miR-200a-3p	[36]
	EVs	Intrarenal injection	Unilateral IRI	Enhanced angiogenesis and cell proliferation, inhibited endoplasmic reticulum stress and apoptosis	-	[37]
	EVs	Intrarenal injection	Bilateral IRI	Improved kidney function, cell proliferation, decreased tubular injury, cell death and fibrosis	miR-let-7a-5p	[38]
	EVs	Intravenous Injection	Unilateral IRI + unilateral nephrectomy	Enhanced angiogenesis, mitigated fibrosis	VEGF (protein)	[39]
	EVs	Intravenous Injection	Unilateral IRI	Increased cell proliferation	-	[40]
	EVs	Intravenous Injection	Unilateral IRI + unilateral nephrectomy	Reduced kidney fibrosis, improved kidney function.	-	[41]
	Exosomes	Intravenous Injection	Unilateral ureteral obstruction	Reduced kidney fibrosis, upregulation of SIRT1, modulation of angiogenesis	-	[42]
iPSCMSCs	EVs	Intravenous Injection	Bilateral IRI	Support to tissue recovery, reduction in necroptosis	Specific protein 1 (SP1) (protein)	[43]
iPSCs	EVs	Subcapsular injection	Bilateral IRI	Reduce cell death and inflammation, protection of mitochondria	-	[44]

Abbreviations: BMMSCs (bone marrow-derived mesenchymal stromal cells); ADMSCs (adipose-derived mesenchymal stromal cells); EPCs (endothelial progenitor cells); KPCs (kidney progenitor cells); HLSCs (human liver stem cells); PDMSCs (perinatal-derived mesenchymal stem cells); iPSCs (induced pluripotent stem cells); iPSCMSCs (iPSC-derived mesenchymal stromal cells); IRI (ischemia-reperfusion injury); AKI (acute kidney injury); EMT (epithelial-to-mesenchymal transition).

The combination of BMMSC-EVs administration with other treatments can also lead to improvements in kidney recovery. The use of pulsed focused ultrasound (pFUS), a short-duration high-intensity pulse of sound waves, led transiently to the release of local inflammatory and chemo-attractive signals [50]. The combinatory treatment showed improved kidney recovery by reversing molecular and histological markers of kidney damage (TIMP1, KIM-1 and NGAL), reducing inflammatory cytokines and increasing cell proliferation in a cisplatin-induced AKI model [24].

### 2.2. Adipose-Derived Mesenchymal Stromal Cells (ADMSCs)

Adipose tissue has emerged as an alternative source of MSCs, requiring minimally invasive procedures and having a higher yield in the MSCs isolation (about 2500-fold higher) [51]. Besides that, ADMSCs have a higher proliferative capacity and are genetically more stable in long-term culture than BMMSCs, which argues for this EV source for therapeutic purposes [52].

Recently, ADMSC-EVs have been described to support kidney recovery by modulating the inflammatory response in a sepsis-induced AKI model. Administration of these EVs led to the increase in Sirtuin 1 expression and reduced NF-kB levels, resulting in animal mortality decrease [30]. Furthermore, in a DOCA-salt-hypertension model, ADMSC-EVs demonstrated the ability to impair CKD progression [28]. Systemic administration of ADMSC-EVs modulated miR-155-5p and members of the miR-200 family that are associated with the TGF-β-dependent epithelial-to-mesenchymal transition (EMT) and fibrosis. Moreover, ADMSC-EVs were capable of attenuating cardiac fibrosis, indicating an important role of EVs in the treatment of complex, multiple organ diseases, such as cardio-renal syndrome [28]. Similar properties were also observed in a renal artery stenosis model associated with metabolic syndrome [33].

New strategies have been applied to improve the therapeutic potential of ADMSC-EVs. The maintenance of ADMSCs under low O_2_ pressure, mimicking physiological conditions, led to increased EV secretion with improved biological effects [29]. Administration of these hypoxic ADMSC-EVs in rats submitted to ischemic injury led to the reduction in oxidative stress by activation of the Nrf2/HO-1 axis, preservation of mitochondrial function and reduction in cell death.

### 2.3. Perinatal-Derived Mesenchymal Stem Cells (PDMSCs)

Several reports have shown that PDMSCs can originate from different parts of the placenta, such as the amniotic membrane, chorionic plate, decidua parietalis and umbilical cord. The effectiveness of PDMSC-EVs to promote kidney cell proliferation, improve kidney function, reduce fibrosis and support angiogenesis has been reported [39,53]. Recently, PDMSC-EVs were encapsulated in a collagen matrix and administrated into the kidney cortex to increase EV stability and allow a more sustained release of EVs [38]. In addition, the creation of an RGD (Arg-Gly-Asp) peptides hydrogel to work as a scaffold for PDMSC-EVs has been reported [39]. These peptides potently bind to integrins present at the EV surface, thereby enhancing and prolonging their bioavailability. These strategies all reported improved protective effects of PDMSC-EVs against kidney injury.

### 2.4. Kidney Progenitor Cells (KPCs)

Kidney tissue presents a progenitor-like cell population that may be responsible for tissue maintenance and regeneration after damage [54,55]. These KPCs have been characterized by a co-expressing of the CD133 and CD24 cell surface markers and have been described to be located in different niches, including the urinary pole of Bowman’s capsule, proximal tubules and inner medulla [55,56,57]. Obtained data using KPCs and the KPC-EVs in AKI treatment demonstrated attenuation of ischemic injury and amelioration of kidney function [34]. Moreover, a miRNomic profile of KPC-EVs revealed a group of miRNAs that regulate several biological processes associated with tissue regeneration (nucleic acid metabolism, transport and regulation of cell growth).

### 2.5. Human Liver Stem Cells (HLSCs)

Human Liver Stem Cells (HLSCs) have been described as a specific population isolated from normal adult human liver, capable of generating insulin-producing islet-like structures and of supporting the regeneration of liver parenchyma [58]. The regenerative property of these cells has also been reported to be applicable to the kidney by using the HLSC-EVs in AKI and CKD models. The results show improved kidney function, enhanced proliferation of tubular epithelial cells, reduction in fibrosis and reversal of CKD progression [36,48].

### 2.6. Induced Pluripotent Stem Cells (iPSCs)

Since the generation of iPSCs by the use of the reprogramming factors (Oct3/4, Sox2, Klf4, c-Myc), different protocols have explored their ability to indefinitely proliferate in a dish and differentiate into almost all the cell types [59]. Such a strategy has been used to generate MSCs from iPSCs, as an alternative source to produce therapeutic EVs [60]. The iPSC-derived MSCs have shown comparable effects in kidney recovery, such as reducing apoptosis and promoting vascularization compared with adult MSCs [44,61]. More recently, EVs isolated directly from human iPSCs were also shown to present therapeutic effects in kidney tissue. Further, subcapsular administration of iPSC-EVs led to a better recovery of the kidney after ischemia-reperfusion injury compared to ADMSC-EVs. The iPSC-EVs were shown to reduce cell death through maintenance of mitochondrial mass, and reduction in tissue damage, macrophage infiltration and oxidative stress [45].

## 3. New Technologies to Improve EVs Therapeutic Application

### 3.1. 3D Culture Systems for EVs Production

The study and production of EVs for kidney therapeutic purposes has mainly been performed in two-dimensional (2D) cell cultures. Although this is a simple and effective approach for collecting EVs, it does not reproduce the spatial organization of the native microenvironment and may alter biomechanical and biochemical cues that influence cell behavior [62]. For instance, 2D models force an apical–basal polarity that cannot be found in vivo for some cell types (e.g., mesenchymal cells). Instead, three-dimensional (3D) models may better mimic physiological conditions by advancing cell–cell interactions, deposition of extracellular matrix (ECM), secretion of growth factors and improving cell morphology (see Table 2) [63,64,65,66,67,68,69]. In a 3D spheroid culture of MSCs, differences in EV composition were observed compared to MSCs cultured in monolayers, including an increase in anti-inflammatory compounds [70,71,72]. Indeed, the secretory behavior and cargo of the EVs depend on cells’ physiological and pathological status and the stimuli from their environment [72], and may provide more physiological-like EVs [73]. Moreover, some studies have shown that 3D culture conditions can increase the amount of EVs produced and improve their biological effects [42,74]; therefore, spatial cell organization has been proposed to be important in the biogenesis and functional outcomes of EVs.

**Table 2:** 3D models and EV therapeutic improvements in the kidney.

3D Models	Advantages	Limitations	EV Improvements for Kidney Treatment	[Ref.]
Hydrogels/Scaffolds	High reproducibility; Use primary or immortalized cells; Allows gradient diffusion; Mimic mechanical forces.	Lack of fluid flow; Simplified architecture; Batch-to-batch variability; Complex imaging analysis.	-	[65]
Spheroid	High reproducibility; Use in microplates; Mimic nutrient/O_2_ gradients; Uniform size.	Simplified architecture; Static condition; Restricted group of cells can generate spheroids.	Increased paracrine secretion immunomodulatory and angiogenic factors, stronger anti-apoptotic and anti-oxidative capacities	[65,66]
Hollow Fibers	Allows culture of large amount of cells; Presence of fluid flow with possibility to collect EV for long periods in culture.	High cost; Requires special equipment.	Increase in the EV production with therapeutic effects (higher protective and anti-inflammatory properties)	[65,67]
Organoids	Realistic micro-anatomy of organs; Can be used to study diseases and developmental processes; Formed from primary cells.	Immature phenotype; Static condition; High cost.	Potential use of urinary EVs as biomakers for kidney disease	[65,68,69]

EVs from kidney organoids may also advance their use in therapy. Urinary EVs mainly originate from kidney epithelium and have been suggested as a method of intra-nephron communication among cells throughout the urinary tract [75]. In effect, urinary EVs derived from tubule cells have been shown to improve kidney recovery after injury [76,77]. Furthermore, EVs derived from KPCs were shown to support the recovery of kidney tissue after cisplatin-induced injury and in glomerular nephritis models [78,79]. Thus, investigation of the therapeutic potential of kidney organoid-derived EVs as a 3D culture system, may be an interesting approach in the treatment of kidney diseases.

### 3.2. Bioengineering Vesicles

The capacity of EVs to transfer bioactive molecules led to the development of new therapeutic strategies based on the modification of their cargo [80]. Different from drug delivery systems such as liposomes, polymer–drug conjugates and polymeric micelles, the use of EVs presents advantages such as structural stability, non-toxicity and low immunogenicity that are relevant issues with synthetic nanoparticles [81].

There are two main approaches for bioengineering EVs: the direct and the indirect method [80]. The direct strategy is based on techniques to load specific molecules directly into isolated EVs. A simple approach is the incubation of lipophilic molecules that can passively be incorporated into the EVs. Such a strategy, however, is limited to the characteristics of the molecule and often presents low loading efficiency. Chemical agents such as lipofectamine (cationic-lipid transfection reagents) have also been used to promote the loading of nucleic acid molecules into EVs. Lipofectamine is considered a “gold-standard” for the delivery of exogenous DNA or RNA into cells, and was explored to promote a direct loading of siRNAs into EVs [82,83]. Despite the incorporation of siRNAs by the EVs, their efficacy was low and the remaining chemical agents can cause toxicity and immunogenicity. On the other hand, a different strategy through electroporation allowed the entrance of hydrophilic and nucleic acids with a higher efficiency, improving EVs’ biological effects [84,85]. However, electroporation has been reported to promote nucleic acid aggregation, which can precipitate together with EVs during the centrifugation process and can compromise the determination of correct efficiency [86]. More recently, a new approach has been designed to load miRNAs into EVs mediated by temperature-controlled co-incubation with miRNAs. For example, there has been a successful demonstration of the incorporation of miR-126, known to support the angiogenic process, into serum-derived EVs [87]. Posteriorly, such EVs were incubated with human endothelial cells (HUVEC), increasing the capacity to induce capillary-like structures. Such an approach reveals the possibility of stably loading specific miRNA cargos to improve their therapeutic effect.

The strategy based on the indirect approach promotes changes in the cell of origin that will posteriorly result in the secretion of EVs enriched with a specific molecule. The cellular reprogramming of MSCs through genetic modifications is one of the main approaches used to improve EVs’ therapeutic effects [88]. The overexpression of proteins such as signaling molecules and transcription factors has been shown to enhance the effect of EVs. In an AKI model, EVs derived from HC-MSCs modified to overexpress the transcription factor OCT4 were shown to reduce the expression of Snail, known to be a trigger of the epithelial-to-mesenchymal transition (EMT) process. Consequently, the administration of EV-modified HC-MSCs led to a better outcome in kidney tissue recovery, improving cell proliferation, abrogating cell death and blocking the initial fibrosis process [41]. Using a different strategy, it was shown that EVs derived from engineered MSCs to overexpress miRNA-let7c were capable of transferring the miRNA into kidney cells and of inhibiting interstitial fibrosis [89]. Furthermore, EVs derived from BM-MSC modified by lentiviruses to overexpress miR-34a suppressed TGF-β1-induced EMT in human kidney tubule cells [90].

The new advances in gene-editing technologies, such as the clustered regularly interspaced short palindromic repeat (CRISPR)/CRISPR-associated protein (Cas) nucleases, have brought the possibility to accurately promote modifications in target genomic loci, allowing correction of mutations, regulating transcription and promoting changes in the epigenome [91]. Due to their biocompatibility and safety properties, EVs have been used as an exciting tool to transiently deliver the gene-editing machinery and successfully promote gene editing in target cells, supporting tissue recovery or treating genetic diseases [92,93,94]. Moreover, the combination of EVs with CRISPR/Cas9 technology led to the development of a highly-sensitive reporter system that permits the tracking and functional analysis of transferred small non-coding RNAs [95].

### 3.3. Bioreactors to Produce EVs

The large amount of EVs required for preclinical and clinical applications is challenging. For clinical tests, a patient would need approximately 100 μg of EV/kg of body weight for each treatment. This requires expansion upscaling and prolonged maintenance of cells [96,97]. Hence, significant numbers of MSC currently are a limiting step to bring EVs to Phase III studies as stable and potent products [98,99,100,101]. The most common system to obtain EVs is a planar culture platform in which cells are expanded in T-flask cultures [102]. This method requires extensive parallel processing, but with low procedure control and a higher risk of contamination or operator errors.

The use of bioreactors has more recently been introduced as a possible solution to maximize the surface area for cell growth and, therefore, increase the capacity and efficiency of EV production. Bioreactor flasks offer a similar format to T-flask cultures, but can be designed to concentrate conditioned medium upon culture. Human embryonic kidney cells were cultured using this system, in the presence of a semi-permeable membrane that allowed continuous diffusion of nutrients and at the same time the accumulation of EVs [103]. Bioreactor flasks require a smaller volume of medium and reduced manipulation of cell culture maintenance, decreasing manual work and making EV collection more cost-effective. Still, the total surface area is often comparable to regular T-flask cultures, and enlarging the surface area would be advantageous to further upscale exosome production. To increase the surface area for adherent cells, scaffolds and microcarriers can be implemented in well-characterized bioreactors [104]. The microcarrier culture systems can promote a homogeneous suspension that improves cell culture robustness and reproducibility [105]. This approach has been used to improve the expansion process of various MSC cultures [106], often combined with dynamic systems for suspension culture such as spinner flasks, stirred-tank bioreactors or wave bioreactors, allowing high-density cultures with low cell damage [107,108,109,110,111].

Furthermore, new dynamic systems that allow continuous perfusion-based cultures have been developed to enable simple media exchange and cell/conditioned medium separation. Such systems can support cultures over an extended period with high productivity. A common example of such systems are hollow-fiber bioreactors, which are 3D systems that use multiple parallel semi-permeable capillary membranes to allow the transfer of nutrients to and waste products from the cells seeded on the extracapillary space [112]. Using this platform, Colao et al. reported the collection of an amount of MSC supernatant equivalent to the yield of 230 conventional T-flasks within 55 days [102]. Moreover, the conditioned medium isolated from this system presented a higher concentration of exosomes with enhanced therapeutic effects in an AKI model compared to exosomes isolated from a 2D culture system [113]. Additionally, some continuous perfusion bioreactors based on microporous matrices (packed bed technology) allowed high efficiency in conditioned medium production during long-term cultures and presented low shear stress rates on cells. Another advantage of perfusion-based bioreactors is that they can be designed to concentrate and collect exosomes within a separate compartment, improving the feeding and harvesting processes [114].

It is worth mentioning that cell expansion in bioreactors may result in phenotypic alterations due to physicochemical differences compared to planar flask cultures, such as cell-to-microcarrier binding, mass transfer and shear stress produced by agitation (impellers) and oxygen sparging [115]. These alterations could affect exosome production, composition and their biological effects. Although these changes remain to be investigated, the use of bioreactors to obtain EVs may still present an efficient tool in the translation of EVs applications to the clinic.

### 3.4. New Technologies for EVs Isolation

The isolation methods currently applied are based on the EVs’ physical and biochemical characteristics, varying through size, density, electrical charges and surface membrane composition (see Table 3) [116,117,118,119,120,121,122,123,124,125,126,127,128,129,130,131,132,133,134,135,136,137,138,139,140,141,142,143,144,145,146].

Classical ultracentrifugation was the first and most commonly used method for EV isolation [117]. This process consists of separation by serial centrifugation cycles, initially removing cell debris and apoptotic bodies and posteriorly producing a pellet with isolated EVs. The limitation of ultracentrifugation is it being a relatively time-consuming, operator-sensitive and low-efficiency method that limits the scale-up process for clinical use. Alternatively, precipitation is often used as a method based on the alteration of solubility of EVs in solution [116]. Despite being an easily performed method, it can also precipitate other particles such as protein aggregates and extracellular protein, resulting in an impure yield. Due to these limitations, novel isolation strategies have been developed to obtain a more pure and EV-specific population.

The different sizes of EVs have been used for designing novel isolation protocols. Examples such as the ultrafiltration system and tangential flow filtration (TFF) use membranes with a defined molecular weight cut-off ranging from 10 to 100 kDa and have been used to isolate EVs from urine-derived cell culture medium [125,126]. Another method is the size-exclusion chromatography (SEC) that separates EVs from different body fluids based on their hydrodynamic volume and molecular size [128]. The combination of two SEC columns (2D SEC) was recently shown to improve the isolation capacity of the different urinary exosome subpopulations [131]. Furthermore, the asymmetrical field-flow fractionation (AsFFF) allows the separation of EVs from plasma contaminants, such as lipoproteins (high-density and low-density lipoproteins), without subjecting the EVs to shear forces [132]. Moreover, the AsFFF efficiency to isolate EVs by size allowed the distinction of a new subpopulation previously defined only by exosomes. Zhang et al. demonstrated the existence of a group of EVs named exomeres (<50 nm) that present distinct composition and biological properties from the small and large exosomes [133].

Another approach in EV isolation is the affinity-based technique, targeting specific proteins at the EV surface membranes. In this respect, immunoaffinity is most broadly used, which relies on the application of antibodies to sort a group of EVs that presents specific surface proteins [84]. A similar strategy can also be applied using single-stranded DNA or RNA oligonucleotides, called aptamers. These structures can recognize a wide range of molecules with high affinity and specificity (such as ions, peptides, nucleic acids and proteins) and have been used to isolate exosomes from urine samples [135,136]. Other techniques, such as ion-exchange, electrophoresis and dielectrophoresis approaches, use the charge of EV membrane components to successfully promote their sorting [130].

**Table 3:** EV isolation methods.

Isolation Method	Principle	EV Type	Sample	Advantage	Limitations	[Ref.]
Centrifugation						
Ultracentrifugation	Density	Exosomes and MVs	CM (conditioned mesdium)/ urine	Isolation of large volumes, cost, simple procedure	Time-consuming, operator-sensitive, damage of EVs, low efficiency, impurity and co-isolation of aggregates	[117,118]
Density gradient ultracentrifugation	Density	Possible subtype isolation	CM/ urine	Purity, better removal of contaminating protein aggregates	Complex procedure, loss of sample	[119,120]
Precipitation						
Precipitation	Solubility	Exosomes and MVs	CM/ urine	Cost, EVs integrity, high yield	May present contamination of polymers, co-isolation of proteins and aggregates	[121,122]
Filtration						
Ultrafiltration	Size	Possible subtype isolation	CM/ urine	Fast and simple procedure, isolation of large volumes, scalable	Filter plugging, low puricity (protein contamination), damage of EVs	[123,124]
TFF (Tangential Flow Filtration)	Size with tangential flow	Possible subtype isolation	CM/ urine	The tangential flow reduces clog of the pore membrane, high yield, large scale, EVs integrity	Contamination of proteins and lipid impurities	[125,126]
Hydrostatic filtration	Size	Exosomes and MVs	Urine	Does not require centrifugation, cost, isolation of large volumes	Combination of other techniques to obtain EVs subpopulations	[127,128]
Size exclusive chromatography (SEC)						
SEC	Hydrodynamic volume or molecular size	Possible subtype isolation	CM/urine	Scalability, EVs integrity, efficiency and purity	Specialized equipment, cost, coisolation of aggregates and proteins, further concentration steps needed	[129,130]
Two-dimensional SEC	Size	Possible subtype isolation	CM/urine	Improve exosome isolation, higher efficiency and purity than SEC	Specialized equipment, sample volume is limited	[129,131]
Filed-flow fraction						
Asymmetrical filed-flow fraction (AsFFF)	Diffusion coefficient	EV subtype isolation	Urine	Less time consuming, possible to isolate EVs from plasma contaminants	Specialized equipment	[132,133]
Affinity						
Immunoaffinity	Antibodies binding	EV subtype isolation, specific exosomes	CM/ urine	Simple and fast procedure, specificity and purity	Non-specific binding, availability of antibodies, costs	[129,134]
Aptamers affinity	Aptamers binding	EV subtype isolation, specific exosomes	CM/ urine	Higher affinity and specificity than immunoaffinity methods	Costs, low yield, prior knowledge of EVs characteristics	[135,136]
Microfluidics						
Multistage filtration	Size	EV subtype isolation	Urine	Efficient, high purity	Low sample capacity	[137,138]
Deterministic lateral displacement (DLD)	Size	EV subtype isolation	CM/urine	Less time consuming	Specialized equipment, scalability	[139,140]
Combination with affinity method	Binding and size	EV subtype isolation, specific exosomes	CM/urine	Allows quantification and characterization of EVs	Specialized equipment, costs	[141,142]
Viscoelasticity-based	Viscoelasticity/size	EV subtype isolation	CM	High purity and faster than DLD method	Specialized equipment	[143,144]
Acoustophoresis	Size	EV subtype isolation	CM	High purity and yield	Need of high-frequency power supply	[145,146]

Furthermore, advances in microfluidics allowed the development of new platforms to efficiently isolate EVs from body fluids on a larger scale [137,138,139,140,141,142,143,144,145,146]. One example is the isolation of urinary EVs by integrating a filtration-based system with a microfluidic platform. The system is based on deterministic lateral displacement (DLD) pillar arrays that allow the separation of particles by size [140]. The particles with a diameter larger than established by DLD geometry are driven outside the system and the smaller particles are carried with the fluid flow, promoting their sorting. The technique was shown to isolate the urinary EVs down to 25 nm sizes with a single-particle resolution, using small volumes and without labeling. Alternatively, Zhao et al. combined the immunoaffinity technique with microfluidic devices to design a platform capable of continuously isolating EVs from human plasma [141]. The system uses magnetic beads coated with antibodies against CD9, CD63 and CD81 to isolate exosomes. In combination with an in situ immunoassay, the device allowed the identification of tumor markers (CA-125, EpCAM and CD24) present in the EVs. Such a strategy points to the potential use of microfluidic devices not only for isolation but also to quantify and perform molecular profiling of the EVs. The use of microfluidics platforms creates the possibility to implement plasma and urine EVs as diagnostic/prognostic tools in the clinic.

## 4. Conclusions

The current advances in EVs studies hold a great promise for the treatment of kidney diseases. The bottleneck in the translation to clinical applications has been widened by the new achievements in the manufacturing and isolation of EVs, allowing large-scale productions that can be easily transported and stored for long periods (long shelf life). Besides, the possibility of bioengineering EVs allows the designing of personalized treatments based on the patient’s kidney pathophysiological aspects. Future challenges concern the standardization of EVs production and the development of methods to control the quality and safety of therapeutic EVs. Such achievements will contribute to translate EVs into the clinic as a diagnostic tool and therapeutic strategy in the treatment of kidney diseases.

## Data Availability

Not applicable.
